# Correction: Intersecting paths: Corporate and green innovation in Chinese firms—A penal cointegration analysis

**DOI:** 10.1371/journal.pone.0318610

**Published:** 2025-01-28

**Authors:** ZhongJi Liu, Dan Hou, R. M. Ammar Zahid

The word "penal" is misspelled in the article title. The correct title is: Intersecting paths: Corporate and green innovation in Chinese firms—A panel cointegration analysis. The correct citation is: Liu Z, Hou D, Zahid RMA (2024) Intersecting paths: Corporate and green innovation in Chinese firms—A panel cointegration analysis. PLoS ONE 19(1): e0295633. https://doi.org/10.1371/journal.pone.0295633
